# The relationship between formal and informal care among Chinese older adults: based on the 2014 CLHLS dataset

**DOI:** 10.1186/s12913-019-4160-8

**Published:** 2019-05-22

**Authors:** Wenyi LIN

**Affiliations:** 1 School of Public Administration, JiNan University, No.601, Huangpu Dadao Xi, Guangzhou, Guangdong Province China; 2 Emergency Management Research Center, JiNan University, Guangzhou, China

**Keywords:** Informal care, Formal care, Chinese older adults

## Abstract

**Background:**

The substitute or complementary effect of formal care on informal care service used by the elderly has been tested in Western countries. However, this effect is excluded from the discussion in the Chinese context. The identification of the relationship between informal care and formal care may imply different directions in policy-making. Thus, this study contributes to understanding the relationship between informal care and formal care among Chinese older adults.

**Methods:**

Using the dataset from the Chinese Longitudinal Healthy Longevity Survey (CLHLS) in 2014, this study uses regression models and instrumental variable (IV) method to examine the impact of formal care on informal care.

**Results:**

The results primarily show that formal care does not substitute informal care among Chinese older adults. In fact, formal care is a supplement to informal care in China.

**Conclusion:**

It is expected that informal care will become less available in the future in China. Thus, policymakers should be concerned about the underdevelopment of formal care for the elderly in China.

## Background

According to the National Bureau of Statistics of China, the Chinese population aged 60 and above accounted for 16.7% of the total population by the end of 2016 (China Civil Affairs’ Statistical Yearbook, 2017). It is widely recognized that elderly care is usually provided by family members in Chinese societies. However, due to the reducing trend of household size and the weakening family support, the availability of family caregivers cannot be taken for granted in the future [12]. In the last two decades, the Chinese governments from the central level to the local level have made much effort to establish a social welfare system to respond to elderly care challenges. Public spending for older adults is increasing, thus policymakers are vigilant about the negative impact of formal care services on informal care mainly provided by family members. The fear that the provision of formal care services might reduce the willingness and practice of informal care is a global issue studied by many scholars [14].

In China, one significant concern is noted with respect to older adults’ care provision, that is, the erosion of *filial piety*. The Asian care system is based on the patrilineal system with an emphasis on Confucian beliefs of filial piety, duty, respect, love and support (Milligan, [13]). The culture of *filial piety* is a key determinant in constructing and reinforcing the responsibility of care and provider of care. In Chinese societies, children who comply with filial piety in daily life are praised by society. Nevertheless, rapid urbanization, increasing aged population, number of nest families and flowing population have resulted in the inability of the majority of young people to practice *filial piety*. Thus, they had to contend with moral condemnation. On the other hand, when policymakers put forward elderly care policies and social service providers deliver elderly care programs, they need to consider the impact of informal care on formal care thoroughly.

The substitute or complementary effect of informal care on formal care service used by the elderly has been tested in Western countries [4]. However, this effect is excluded from the discussion in the Chinese context. The identification of the relationship between informal care and formal care may imply different directions in policy-making. This study makes a contribution to understanding the following two questions: first, does formal care substitute or complement informal care? Second, what factors could predict the utilization of formal and informal care?

## Literature review

A majority of available international studies indicate the complexity of the relationship between informal care mainly provided by families,and formal care provided by professionals [1, 3–5, 10, 15, 19]. The studies on the relationship between informal and formal network care services have developed two important models to understand such interaction. The first model is called the *dual specialization model* and suggests a coordinated interaction between informal and formal networks when each assumes responsibilities to which they are most suited (Wacker and Roberto, [23]). Generally, an informal network is considered suitable to address the unplanned and unscheduled needs of the elderly, whereas formal network expertise is used to provide scheduled and structured care service (Wacker and Roberto, [23]). By contrast, the *supplemental model* considers a supplementary relationship between informal and formal networks (Wacker and Roberto, [23]). The failure of an informal network to meet the needs of a care recipient can be complemented by the availability of a formal network (Wacker and Roberto, [23]).

Informal care from the family is a significant component that is associated with care services for the elderly. Two studies conducted in Europe and the US supported the conclusion that informal care from the elders’ children can provide a good substitute for formal care service in terms of home care and health care services [7]. However, the study in Europe indicated that the alternative effect was likely to wane as the level of disability of the elders increased [24]. Wacker and Roberto [23] perceived families with care provision for the elderly as gatekeepers when old people use formal care service. In other words, informal care seems to have a negative effect on the access of elders to formal care support in the community.

In China, informal care is usually provided by family members, relatives or friends or neighbors in the home of the person who needs assistance in activities of daily living (ADL) and instrumental activities of daily living (IADL). Under the Chinese socio-cultural context, cultural norms in terms of *filial piety* may unsurprisingly produce an effect on the elderly’s access to social services. Qian [17] conducted a telephone survey that involves 963 Hong Kong Chinese whose parents were alive in 2002 and analyzed the impact of *filial piety* on care model preference for this group of people. The result of the aforementioned survey presented a strong negative impact of *filial piety* on the desire and preference for government support for the elderly. Accordingly, the effect was substantially significant among people aged 40 years and above. Another study conducted in Shandong Province determined that 24 older adults highlighted *filial piety* when receiving family care, although they did not require their children to assume the responsibility of filial piety in daily life [2]. These adults felt delighted as long as their children respected them. Nevertheless, the former did not want to cause trouble for their children. Thus, when their children did not have time to provide care service, they would accept the arrangement of placing them in nursing homes. It implies formal care was not the first preference for these older adults.

The substitute or complementary effect of informal care on formal care service has not been fully discussed in the Chinese context. In order to formulate effective long-term care policies, it is necessary to understand the relationship between informal care and formal care. The existing studies for Chinese older adults have recognized the negative effect of formal care on informal care, but ignored the problem of variable endogeneity [11]. In addition, since the data is not from the national dataset, and most studies are based on convenience samples, the existing studies have the problem of generalizing the findings to all older adults in China. This study aims to resolve the problem of variable endogeneity and the problem of generalizability. Thus, through analyzing the effect of formal care in terms of home-based formal care services and health formal care services on informal caregiving, this study makes a contribution to understanding the relationship between informal and formal care in the Chinese society where emphasizes informal care. Moreover, this study makes an effort to deal with the casual relationship between informal and formal care through using the instrumental variable (IV) method.

## Method

### Data sources and participants

The data used in this study mainly came from the Chinese Longitudinal Healthy Longevity Survey (CLHLS) in 2014, which is a national survey. A total of 7192 adults aged 47 and above participated in CLHLS in 2014. This dataset includes 2369 older adults aged 65–79 and 4738 oldest-old aged 80+.^1^ The secondary analysis of data from CLHLS did not require ethical approval.

The sample includes older adults aged 60 years and above who have been invited to answer the survey based on their voluntary participation (*N* = 1697). Among the participants, 838 were from urban areas and 859 were from rural areas. This study uses regression models and IV method to examine the impact of formal care on informal care and to identify the factors predicting service use of informal care and formal care.

### Variables and measures

#### Dependent variable

Informal care is a dependent variable in this study. In the 2014 CLHLS survey, informal care was measured as the total number of hours the respondent’s children, grandchildren, and their spouses help her/him last week.

#### Independent variable

The primary independent variable is receiving formal care in terms of home-based formal care and health formal care. In the 2014 CLHLS, a question: “Who is the primary caregiver when you need assistance in above bathing, dressing, toileting, indoor transferring, continence, and eating?” was proposed for the respondent to identified whether she/he had received home-based formal care. If the respondent said social services or housekeepers were the primary caregivers, then the respondent was identified as a receiver of formal care. Otherwise, if family members, relatives, or friends or neighbors were the primary caregivers, then the respondent was identified as a receiver of informal care. Thus, the home-based formal care variable was a dummy variable with home-based formal care = 1 and informal care = 0. Another question measuring health formal care in the CLHLS was associated with yearly inpatient expenditure: “How much did you spend on inpatient costs last year?” The total expenditure for yearly inpatient costs was calculated to measure the health formal care utilization.

Cognitive status was measured through a mini-mental state examination assessing the respondent’s orientation, memory, attention and calculation, and language ability. After that, the respondent’s cognitive status was evaluated by three levels from 1 to 3, with 1 indicating good cognitive status, and 3 indicating bad cognitive status.

The respondent’s basic characteristics were included in the analysis to control for variance in the use of informal care based on gender, age, marital status, income, and residence area. Gender was dummy-coded into groups, with male = 0 and female =1. hedge variable was a continuous variable, calculated based on the reported birth date of each respondent. Marital status was dummy coded into married or not married (married =1, unmarried =0). Income was a continuous variable, calculated based on the reported income per capita of the respondent’s household last year. Residence area was dummy coded into urban or rural (urban = 1, rural = 0).

Another two variables measuring the need and the availability of community-based social services were included in the analysis for predicting the use of formal care. In the CLHLS, the respondent was asked what kind of eight social services were available in her/his community and what kind of these eight social services did she/he expect to be provided in the community. Eight social services included personal daily care services, home visits, psychological consulting, daily shopping, social and recreation activities, human rights consulting services, health education, and neighboring relations. The number of community-based social services was calculated to measure the availability and the need for community-based social services.

#### Iv

Two IVs predict formal care use. The first IV is the ADL score, and the second IV is the IADL score. The existing studies have identified ADL and IADL are the strongest predictors for the utilization of formal care [4, 21, 22]. In the CLHLS, there were six indicators assessing the participant’s ADL capacity: bathing, dressing, toileting, indoor transferring, controlling urination and bowel movement, and feeding. Eight indexes were associated with IADL capacity: visiting neighbors, shopping, cooking, washing, walking, lifting, crouching and standing up three times, taking public transportation. Each indicator or index was coded from 1 to 3, with 1 indicating the respondent could finish the task independently and 3 indicating the respondent could not finish the task, making the minimum score 6 and the maximum 18 for ADL and the minimum score 8 and the maximum 24 for IADL. Cronbach’s alpha test was used to analyze the internal consistency of items relevant to ADL and IADLS. The summed index had a Cronbach’s alpha of 0.875 for ADL and 0.954 for IADL.

### Data analysis

Descriptive statistics were provided to understand the informal and formal care, as well as the basic information of the older people. Afterward, regression models were developed to examine the factors predicting informal or formal care service use and the effect of IVs on formal care. And then a two-stage model was adopted to test the effect of formal care on informal care. All predicting variables were simultaneously included in these models. The statistical software package SPSS 24.0 was used for all data analysis.

## Result

Table 1 presents the respondents’ descriptive data. The average hours of informal care received by older adults were 41.54. Among 1697 older adults, 92.90% received assistance from informal caregivers and thought informal caregivers were their primary caregivers in need.

**Table 1:** Characteristics of respondents (*n* = 1697)

	Mean or percent	Standard deviation	Minimum	Maximum
Hours of informal care	41.54	57.78	0	720
Primary caregiver (informal caregiver)	92.90%	–		
Yearly inpatient expenditure	3919.43	12,253.33	0	More than 100,000
Gender (male)	38.50%	–		
Age	90.90	10.33	61	116
Marital status (unmarried)	72.60%	–		
Residence (rural)	50.60%	–		
Income per capita of the household yearly	35,474.89	31,953.18	0	More than 100,000
ADL	9.83	3.61	6	18
IADL	19.38	5.81	8	24
Cognitive status	0.92	0.86	0	2
Not impaired	41.20%			
Partially impaired	25.60%			
Impaired	33.20%			
Number of available social services in the community	1.56	1.88	0	8
Number of expected social services in the community	5.10	3.13	0	8

In the sample, female respondents outnumbered male respondents (38.50%). The respondents had an average age of 90.90 years (SD = 10.33), and the majority of these older adults (72.60%) were in the unmarried status. 50.60% of the sample were from rural areas, slightly more than the urban sample. The income per capita of the respondent’s household was 35,474.89 *Renminbi* (RMB) yearly. The mean of ADL was 9.83 (SD = 3.61) and IADL was 19.38 (SD = 5.81). 33.20% of older adults were cognitively impaired, and 25.60% were partially impaired. The average number of available social services in the community was 1.56, and the average number of expected social services was 5.10. The two figures indicate the availability of social services for older adults in China remains low and older adults expect more social services provided in the community.

Table 2 presents the odds ratio associated with home formal care utilization. The dependent variable in this equation is the use or nonuse of a formal caregiver as the primary caregiver. The independent variables consist of all the exogenous variables included in the regression model and two IVs (ADL scale scores and IADL scale scores). The two IVs are significantly associated with formal care utilization (both *p* < 0.01). Age, marital status, residence status, income, and the number of available social services in the community significantly influence the utilization of formal care. Concretely, younger and unmarried older adults are more likely to use formal care as primary care. Older people living in the urban area and in the community where could not provide more social services are more inclined to utilize formal care. The measure of needing formal care, namely the number of expected social services in the community, does not affect the utilization of formal care.

**Table 2:** The effect of ADL and IADL on receiving home-based formal care among older adults (*n* = 1697)

	Odds ratio	95% CI	*p*-value
Constant	96.220		0.001
Gender ^a^	0.707	0.707–0.444	0.146
Age	0.929	0.929–0.906	0.000
Marital status ^b^	0.161	0.161–0.080	0.000
Residence status ^c^	2.925	2.925–1.752	0.000
Income per capita yearly	1.000	1.000–1.000	0.000
ADL	1.110	1.110–1.032	0.005
IADL	1.093	1.093–1.024	0.008
Cognitive status	0.769	0.769–0.575	0.077
Number of available social services in the community	0.765	0.765–0.691	0.000
Number of expected social services in the community	0.961	0.961–0.891	0.301

*Note:* ${}^{\chi^2}$ =237.765, df = 10,*p* < 0.000,Nagelkerke R2 = 0.340. ^a^ Referent is female. ^b^ Referent is married. ^c^ Referent is urban sample

Table 3 shows the estimates of hours of informal care utilization using both the regression and IV models. In the ordinary least square (OLS) model, the utilization of formal care reduces the hours of informal care utilization (B = -29.524, *p* < 0.01). This result suggests the formal care may substitute informal care. To obtain a more definitive answer to the relationship between informal and formal care, an IV model was estimated. In the IV model, on the contrary, the utilization of formal care increases the likelihood of the hours of informal care utilization (B = 476.068, *p* < 0.05). It seems the utilization of formal care cannot substitute informal care. One possible explanation is that the existing social services could not fulfill the needs of older adults who need care resources, thus, those who are more impaired in the abilities of ADL and IADL need formal care on the one hand, and need more informal care on the other due to the scarce of formal care. Together, the two results indicate that the utilization of formal care does significantly affect hours of informal care utilization, although the effects are not the same. In the OLS model, the important factors influencing hours of informal care utilization include the utilization of formal care, age, marital status, residence status, income per capita of respondent’s household yearly, and cognitive status. In the IV model, utilization of formal care, gender, age, marital status, residence status, income per capita of respondent’s household yearly, and the number of available social services in the community significantly affect the hours of informal care utilization. The measures of needing formal care, namely the number of expected social services in the community, does not affect the hours of informal care utilization.

**Table 3:** The effect of home-based formal care on receiving informal care among older adults (*n* = 1697)

	OLS^+^		IV^++^ (2SLS)	
	Beta	St. Beta	Beta	St. Beta
Constant	− 49.478**		− 397.771**	
Primary care^a^	−29.524**	− 0.128	476.068**	2.066
Gender^b^	3.106	0.026	17.358*	0.146
Age	0.801**	0.142	2.787**	0.496
Marital status^c^	−8.669*	−0.067	47.070**	0.362
Residence status^d^	11.243**	0.097	−16.181*	−0.139
Income per capita yearly	9.489E-5^+++^*	−0.052	− 0.001**	− 0.420
Cognitive status	5.729**	0.0085	3.366	0.050
Number of available social services in the community	0.775	0.025	9.615**	0.305
Number of expected social services in the community	−0.861	−0.037	0.520	0.028
*R* ^2^	0.081		0.031	
F-statistic	16.209**		5.954**	

*Note:* ^+^ordinary least square, ^++^instrument variable, ^+++^ 0.00009489,*p < 0.05, **p < 0.01

^a^Referent is formal care. ^b^ Referent is female. ^c^ Referent is married. ^d^ Referent is urban sample

Table 4 illustrates the estimates of health formal care in terms of yearly inpatient expenditure. The two IVs are significantly associated with yearly inpatient expenditure (both *p* < 0.01). Table 5 shows the estimates of hours of informal care utilization using both the OLS and IV models. Similar to the effect of home formal care on informal care, both OLS and IV models indicate that inpatient expenditure has a significant relationship with hours of informal care utilization.

**Table 4:** The effect of ADL and IADL on receiving health formal care among older adults (*n* = 1607)

	Beta	St. Beta	t
Constant	6983.304*		2.013
ADL	378.370**	0.110	3.369
IADL	184.714**	0.087	2.332
Gender^a^	−38.835	−0.002	− 0.057
Age	− 129.945**	− 0.108	−3.482
Marital status^b^	2190.101**	0.079	2.632
Residence status^c^	1471.026*	0.059	2.342
Income per capita yearly	0.065**	0.162	6.349
Cognitive status	− 645.822	−0.045	−1.640
Number of available social services in the community	−31.642	−0.005	− 0.182
Number of expected social services in the community	− 246.531*	−0.062	−2.472
*R* ^2^	0.081		
F-statistic	13.829**		

*Note:* *p < 0.05, **p < 0.01

^a^Referent is female. ^b^Referent is married. ^c^ Referent is urban sample

**Table 5:** The effect of health formal care on receiving informal care among older adults (*n* = 1607)

	OLS^+^		IV^++^ (2SLS)	
	Beta	St. Beta	Beta	St. Beta
Constant	−56.296**		− 124.082**	
Yearly inpatient expenditure	0.0004**	0.088	0.010**	
Gender^a^	3.367	0.028	1.782	0.015
Age	0.886**	0.157	1.518**	0.269
Marital status^b^	−8.047*	−0.062	−26.255**	−0.201
Residence status^c^	8.385**	0.072	−8.668	−0.074
Income per capita yearly	2.625E-5^+++^	0.014	−0.001**	−0.332
Cognitive status	5.786**	0.085	4.885	0.072
Number of available social services in the community	−1.035	−0.032	0.250	0.008
Number of expected social services in the community	0.828	0.044	2.745*	0.147
R^2^	0.075		0.035	
F-statistic	14.019**		6.298	

*Note*: ^+^ordinary least square, ^++^instrument variable, ^+++^ 0.00002625,*p < 0.05, **p < 0.01

^a^Referent is female. ^b^Referent is married. ^c^ Referent is urban sample

## Discussion

Findings of this study indicate that factors associated with formal care utilization include the respondent’s age, marital status, income status, ADLs and IADLs, the availability of social services in the community. The most important finding of this study identifies the relationship between informal care and formal care. Unlike the findings from western countries [14, 21, 22], this study indicates the utilization of formal care may affect the hours of informal care utilization, and the relationship of formal care and informal care is positive. In other words, the relationship between informal and formal care is complementary in China, which is similar to the findings conducted by Chen et al.[5] in Japan.

In recent years, in order to ease the public financial pressure, European countries attempt to promote informal care [4]. In China, the increase in the proportion of the aged population with care needs leads to the growth of public health care spending, so the Chinese government encourages informal caregiving to prevent the increasing cost of formal care. Additionally, the Chinese government is afraid that the provision of formal care will make the Chinese traditional filial piety weaken. However, along with the increasing old-age dependency ratio^2^[6] as well as the increasing number of disabled elderly^3^ [20], it is expected that informal care will become less available in the future in China. Thus, policymakers should be concerned about the underdevelopment of formal care for the elderly in China, and the barriers of accessing formal care facilities and services which have been identified in European countries [8, 18].

Regarding social services with accommodation for older adults, by the end of 2016, there were 140 thousand welfare institutions and facilities for older adults who need institutional care. Concretely, 29 thousand registered welfare institutions, 35 thousand community-based welfare institutions and facilities, and 76 thousand community-based mutual aid facilities totally providing 7.302 million beds for older adults (China Statistical [6]). It indicates one bed is provided for around 32 older adults (60+). As regards social services without accommodation, 1.828 thousand public institutions, 19 thousand legal aid centers, 70 thousand rights safeguarding institutions, 54 thousand elderly school, and 359 thousand activity rooms were provided for older adults who need community-based social services (ibid.) All these resources and services were allocated among 230 million older adults. Predictably, formal care resources and services are limited in China. One more problem is the limited public financial support for formal care. Only 23.554 million older adults received the elderly subsidy, 405 thousand received nursing care subsidy, and 2.829 million received the elderly care service subsidy (ibid.). All these figures illustrate the Chinese government should provide more resources and services to develop formal care system in the next decades when older adults have higher demands for long-term care.

Additionally, the findings of this study illustrate the integrated care model including informal caregivers and professionals is suitable in the long-term care system of China. As asserted by Kerpershoek [9] and Powell [16], the combination of informal care and formal care in the elderly care system will be helpful to older adults who have care needs.

One more noticeable finding is the relationship between residence status and receiving care services. Compared with the rural sample, older adults living in urban areas are more inclined to receive more formal and informal care (See Table 2, OLS model in Table 3, Table 4, and OLS model in Table 5). Nevertheless, in the IV regression model of Table 3, whereas controlling for the endogeneity of informal care rural older adults are more likely to receive more informal care than urban older adults. The possible interpretation is that few formal care services are available in rural areas, thus, rural older adults tend to receive informal care when they need assistance dealing with ADLs and IADLs. It implies both informal and formal care services have urban-rural differences in China. And more formal care services should be delivered in rural areas to respond to older adults’ needs for long-term care services.

This study has a limitation that should be noted. Some variables relating to caregivers were not used in the regression and IV models due to the concern that the variables might be endogenous for hours of informal care utilization. Although this is a weakness, it is noted that the dataset is a national dataset which makes the findings more generalizable.

## Conclusion

We can conclude that formal care including home-based formal care and health formal care supplements rather than substitute informal care in China. Considering that informal care will become less available in the further and formal care is underdeveloped in China, especially in rural China, the Chinese government at all levels should make policies for developing formal care services, and mobilize social resources to establish long-term care facilities and programs.

## Abbreviations

ADL: Activities of daily living
CLHLS: Chinese Longitudinal Healthy Longevity Survey
IADL: Instrumental activities of daily living
IV: Instrumental variable
OLS: Ordinary least square
RMB: *Renminbi*

## Footnotes

1 CLHLS is conducted by the Center for Healthy Aging and Development Studies, and the National School of Development of Beijing University. See http://opendata.pku.edu.cn/dataverse/CHADS for further details.

2 The old-age dependency ratio was 15.0% in 2016.

3 The number of disabled elderly was around 37 million in 2013, accounting for 19% of the aged population (60+).
